# Integrating science‐based and local ecological knowledge: a case study of mangrove restoration and rehabilitation projects in the Philippines

**DOI:** 10.1111/disa.12630

**Published:** 2024-06-05

**Authors:** Gian Powell B. Marquez, Ronald Dionnie Olavides

**Affiliations:** ^1^ College of Global Liberal Arts, Ritsumeikan University Japan; ^2^ BlueNomads.Org Survey Philippines Philippines

**Keywords:** disaster risk reduction, ecosystem approach, local ecological knowledge, mangrove, Philippines, resiliency, science‐based ecological knowledge

## Abstract

Mangrove forest is an ecosystem‐based solution for disaster risk reduction in the Philippines, but its historical deforestation has hampered its capacity to protect coastal communities. With the increasing occurrence of storm surge in the Philippines, mangrove reforestation projects have received renewed attention, but many have failed. Community participation was deemed to be essential in those projects that did well. Hence, this paper examines successful mangrove restoration and rehabilitation projects in the Philippines to find out how community participation contributed to the accomplishments. The study found that while the transfer of science‐based ecological knowledge from project managers to the community is an important factor in ensuring successful initial planning and implementation, its integration into existing local ecological knowledge—‘localisation’ of science‐based ecological knowledge or hybrid ecological knowledge formation—helped to facilitate long‐term community‐based mangrove management beyond project duration by empowering community members and enabling project acceptance and ownership. Still, continuous local institutional support is a necessary anchor for community resilience.

## 要旨

マングローブ林は、フィリピンにおける防災のための生態系ベースのソリューションだが、歴史的な森林伐採により、沿岸地域社会を保護するマングローブ林の力が弱まっている。フィリピンで高潮の発生が増加しているため、マングローブ再植林プロジェクトが再び注目を集めたが、多くは失敗に終わった。マングローブプロジェクトの成功には地域社会の参加が不可欠であると考えられていた。そこで筆者らは、コミュニティの参加がこの成功にどのように貢献したかを調べるために、フィリピンで成功したマングローブの修復および再生プロジェクトを調査した。科学に基づいた生態学的知識 (SEK) をプロジェクト マネージャーからコミュニティに移転することが、初期計画と実装を確実に成功させる上で重要な要素である一方で、SEK と既存の地域の生態学的知識 (LEK) の統合、つまり SEKまたはハイブリッド生態学的知識 (HEK) の形成の「ローカライズ」がコミュニティに力を与え、プロジェクトの受け入れと所有権を可能にすることで、プロジェクト期間を超えた長期的なコミュニティベースのマングローブ管理を促進するのに役立っていることが明らかになった。継続的な地域の制度的支援は、依然として地域社会の回復力に必要な基盤である。

## 摘要

红树林是菲律宾基于生态系统且减少灾害风险的解决方案，但历史上的森林砍伐削弱了其保护沿海社区的能力。随着菲律宾风暴潮频繁发生，红树林恢复造林项目重新受到关注，但许多项目都以失败告终。社区参与被认为是对于红树林项目的成功至关重要。因此，我们研究了菲律宾成功的红树林恢复和重建项目，以了解社区参与如何促成这一成功。我们发现，虽然将基于科学的生态知识 (SEK) 从项目经理转移到社区是确保成功进行初步规划和实施的重要因素，但将 SEK 与现有的当地生态知识 (LEK) 相结合——SEK 的“本土化”或混合生态知识 (HEK) 的形成——有助于通过赋予社区权力并使项目接受和拥有权来促进项目持续时间之外的长期社区红树林管理。尽管如此，当地机构持续的支持仍然是社区恢复力的必要支柱。

## 1 INTRODUCTION

The Global Climate Risk Index ranked the Philippines fourth in the list of countries most affected by weather‐related events from 2000 to 2019 (Eckstein, Künzel, and Schäfer, 2021, p. 46). This is due to its geographical vulnerability to a rising sea level and intensifying typhoons, bringing stronger storm surge and heavier precipitation, respectively (Cardenas et al., 2015, p. 2844; Cinco et al., 2016, p. 4648). The disastrous weather‐related impacts are further magnified by the large concentration of people living in low‐lying coastal areas (Cinco et al., 2016, p. 4638). To improve coastal community protection, the building of artificial coastal defences such as seawalls, revetments, and dykes is commonly recommended. However, these artificial coastal structures are both economically and ecologically expensive (del Valle et al., 2020, p. 265), making ecosystem‐based protection a more affordable and sustainable approach to disaster risk reduction (Temmerman et al., 2013, p. 79).

**FIGURE 1:**
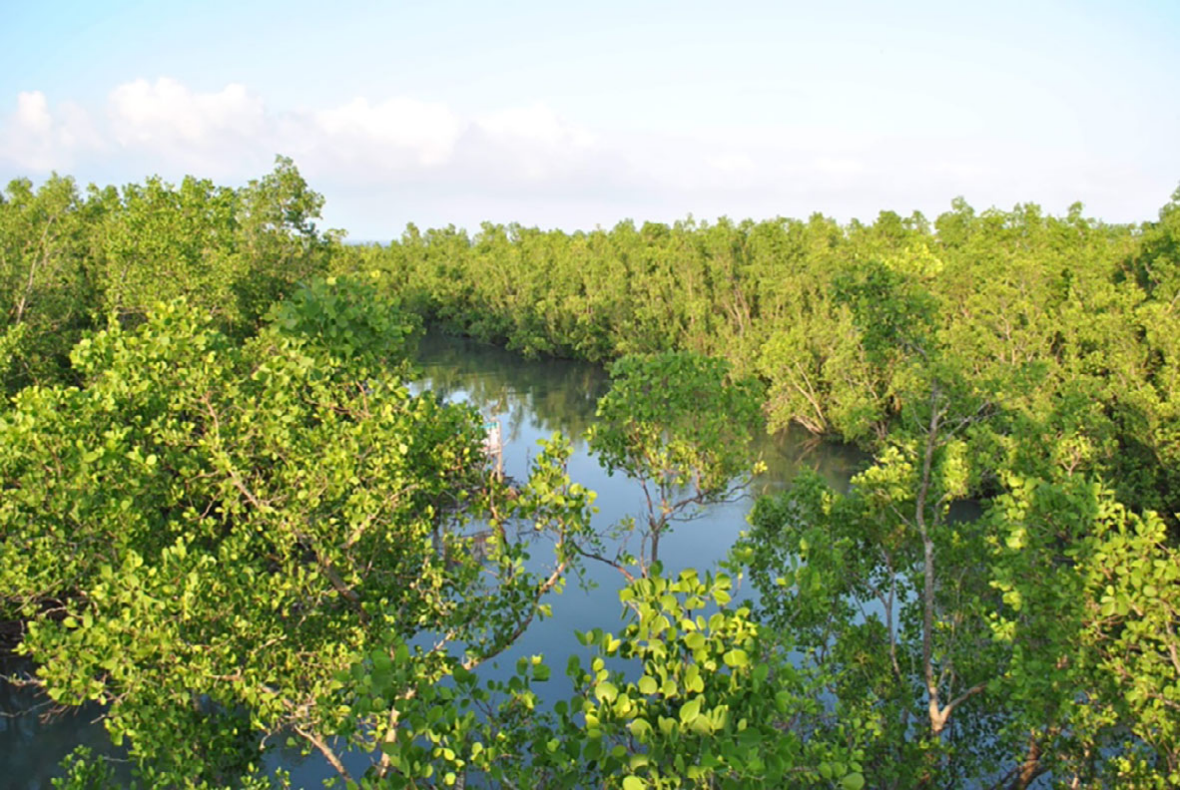
The Silonay Mangrove Conservation Eco‐Park. **Note:** this community‐based mangrove rehabilitation, conservation, and ecotourism park in Calapan City (Oriental Mindoro, Philippines) is recognised as one of the best community‐managed, marine‐protected mangrove areas in the Philippines and as a model for ecosystem‐based adaptation to climate change. The site is also a model of an effective community‐led rehabilitation and conservation initiative by a people's organisation in partnership with a local government unit, non‐governmental organisations, and the private sector. **Source:** Ronald Dionnie Olavides/Conservation International Philippines.

In the Philippines, mangrove forests (see Figure 1) naturally grow along the country's muddy (middle to upper) intertidal zones. They serve as flood buffers, breeding and feeding areas of marine fauna, and natural protection against coastal erosion (Sandilyan and Kathiresan, 2015, p. 94; Carrasquilla‐Henao et al., 2019, p. 1). Despite the potential of mangrove forests as ecosystem‐based protection against disasters, most mangrove areas in the Philippines have been deforested, leading to recorded forest loss of 51.8 per cent in 2010 (Long et al., 2014, p. 268).

This mangrove deforestation was facilitated by overexploitation and government‐initiated fishpond conversion in the 1970s. The overharvesting of mangrove forest was driven by increasing demand for timber, firewood, or charcoal production (Primavera and Esteban, 2008, p. 346). While this provided the surrounding community with quick cash, there was inadequate awareness of the indirect benefits of ecological services (Camacho et al., 2020, p. 19). Moreover, the promise of food security, global competitiveness, and economic growth upon industrialisation of aquacultural sectors drove mangrove deforestation for fishpond expansion throughout the Philippines (Dieta and Arboleda, 2004, p. 155).

Recognising the importance of mangrove forest, as early as 1984, the first internationally‐backed mangrove reforestation project was implemented in the Central Visayas region of the Philippines through the development assistance fund of the World Bank (Primavera and Esteban, 2008, p. 347). But the serious need to rehabilitate and restore mangrove forest only received renewed interest after the devastation wrought by Typhoon Haiyan (Super Typhoon Yolanda) in November 2013. Haiyan caused deadly storm surges along its path, particularly in the coastal communities of Tacloban City (Leyte, Philippines), which could have been minimised if a robust and diverse mangrove forest was present (Villamayor et al., 2016, pp. 2, 13).

Given this current situation and anticipation of future climate change, it is only logical to integrate mangrove restoration and rehabilitation projects in the country as part of a low‐cost and sustainable disaster risk reduction strategy (Blankespoor, Dasgupta, and Lange, 2017, p. 486). However, many mangrove rehabilitation and restoration projects that have been implemented since the 1980s failed (with only 10–20 per cent mangrove survival) despite the reported ballooning cost (from USD 100–500 per hectare) (Primavera and Esteban, 2008, p. 356). Similarly, failed mangrove projects have been observed in Indonesia despite a significantly higher restoration cost (USD 3,900 per hectare) than in the Philippines (2012 estimate of USD 2,000–3,000 per hectare) (Primavera et al., 2012, p. 49; World Bank, 2022, p. 5).

Regardless of the availability of funds, the high failure rate was attributed by many mangrove experts to inadequate adoption of science‐based ecological knowledge (SEK) (Samson and Rollon, 2008, p. 239; Camacho et al., 2020, p. 19). Yet, the inclusion of other knowledge systems, particularly local ecological knowledge (LEK) and traditional ecological knowledge (TEK), as complements to SEK for effective ecological restoration and conservation, has been recognised in the Paris Agreement (no. 5 of article 7; UNFCC, 2015) and the Convention on Biological Diversity's (2020) Aichi Biodiversity Targets (no. 18).

Hence, while adoption of SEK is deemed essential for successful project planning and implementation within project duration, it is important to understand how it has been received and transformed by the participating community, together with its interaction with LEK and TEK, in local ecological management after project duration. This is to comprehend why many projects require community participation, aside from SEK adoption, to be successful in the long term.

Therefore, this paper first defines knowledge systems used in ecological restoration and rehabilitation and explores the dynamic interaction that led to the long‐term success of projects in the Philippines. Next, it introduces a case study of mangrove restoration and rehabilitation projects in the country and identifies key actors and their roles within and beyond project duration. Subsequently, it determines how these key actors used available ecological knowledge to facilitate the short‐ and long‐term success of mangrove restoration and rehabilitation projects.

## 2 ECOLOGICAL KNOWLEDGE SYSTEMS AND THEIR DYNAMIC INTERACTION

Different ecological knowledge systems are important in ecosystem restoration and rehabilitation. In this section we define and differentiate local ecological knowledge, traditional ecological knowledge, science‐based ecological knowledge, and hybrid ecological knowledge (HEK) in the context of how they are being generated, validated, and transmitted. The distinction is necessary to determine how they are interacting within the context of mangrove restoration and rehabilitation projects.

LEK is the ‘knowledge held by a specific group of people about their local ecosystems’, which ‘derive[s] from more recent human‐environment interactions rather than being embedded in deeper cultural practices’ (Raymond et al., 2010, p. 1768). LEK is formed through the direct non‐systematic interaction of people with their local ecosystem. The non‐systematic way of knowing of LEK, as well as the reliance on experiences with nature, made such knowledge validation and evolution slow. Conversely, TEK is defined by Berkes (2012, p. 7) as ‘a cumulative body of knowledge, practice, and belief, evolving by adaptive processes and handed down through generations by cultural transmission, about the relationship of living beings (including humans) with one another and with their environment’.

Comparing the two, LEK can be regarded as a foundation of TEK formation whereas TEK is distinguishable from LEK in terms of how ecological knowledge is preserved and transmitted. TEK is preserved through intergenerational experiential validation and transmitted within the community across generations by embedding ecological knowledge within cultural practices and beliefs. Hence, while both LEK and TEK have undergone similar knowledge validation—through direct human–environment interaction, under the slow process of natural ecological change—the hold of TEK in the community is stronger owing to its integration into a community's culture and tradition. This process of TEK formation ‘institutionalises’ LEK within the community, which serves as one of the intergenerational ‘policies’ on how to interact with nature and influence a world view (Berkes, Colding, and Folke, 2000, pp. 1256–1257). ‘Institutionalising’ LEK through TEK formation ensures that LEK formed by a certain group of people is shared and transmitted among other members of the community. However, while embedding LEK within traditional practices and beliefs has its benefits, its flexibility for validation and improvement decreases. This is because of the changes needed not only with respect to ecological knowledge, but also the culture, tradition, or beliefs where it is embedded. But it is important to emphasise that the foundation of TEK is LEK, and LEK generation is conducive to changes in accordance with the environmental condition. Consequently, a community will adapt via LEK revalidation, which may eventually lead to TEK revision if slow environmental changes remain and thus allow the newly formed LEK to be valid for a long period (Berkes, Colding, and Folke, 2000, p. 1,257).

Given the current impact of global warming, though, which spawns abrupt and constant changes to the natural environment, maintaining a valid LEK within a certain amount of time to permit TEK formation can be difficult. The increasing occurrence of extreme weather events and changing weather patterns, as well as the anthropogenic destruction of other connected ecosystems, bring fast‐changing environmental conditions that result in unstable human–environment interaction and hence, constant LEK generation and revalidation. Nevertheless, the foundation of LEK is based on continuous observation and experiencing nature, which while unsystematic, build reliability through the repeated use of LEK in the community (Sidik, 2022, p. 286).

Ecosystem restoration and rehabilitation previously aimed to promote environmental conservation of protected areas. However, with the growing number of stakeholders, the goal of protected areas evolved beyond environmental protection and resulted in the inclusion of the three tenets of sustainability: environment; society; and economy (Watson et al., 2014, p. 67). Achieving sustainable ecological management requires, therefore, recognition and engagement of multidisciplinary, interdisciplinary, and multi‐system ways of knowing to find the most suitable approach to address the unique but interconnected needs of stakeholders in terms of environmental protection, societal well‐being, and economic benefits across generations (Palomo et al., 2014, p. 185, 186; Watson et al., 2014, p. 71).

While SEK has played a major role in the successful ecological management of many protected areas (Cash et al., 2003, p. 8,089; Watson et al., 2014, p. 71), LEK has remained an important knowledge system in supplementing, if not substituting, science‐based methodology in the field of ecology (if empirical data are lacking) because of its economical, practical, and holistic approaches to gathering data, such as in the case of determining species abundance and diversity (Braga‐Pereira et al., 2021, p. 753), which is important for ecological conservation and management (Cook et al., 2014, p. 2). Accordingly, the practitioners of LEK were reported to be receptive to SEK, but they use it in a way which fits with their local needs (Reid, Williams, and Paine, 2011, p. 10). This ‘localisation’ of SEK in accordance with LEK leads to HEK. The generalisable nature of scientific knowledge as defined by the National Academies of Sciences, Engineering, and Medicine (2019, p. 31) as ‘rules that remain true even if the context of a separate study is not entirely the same as the original’ allowed this harmonious assimilation of SEK in the context of LEK. Inversely, the compatibility of LEK with SEK demonstrates the validity of LEK claimed by many scholars. In this paper, though, HEK is used to delineate indigenous LEK from ‘localised SEK in accordance with LEK’ for a clearer comparison in the discussion, but we do not aim to establish a categorisation and establish a border for the flow and transformation of knowledge among the categories (LEK, TEK, HEK, traditional hybrid ecological knowledge (THEK), and SEK). In the case of ‘institutionalising’ HEK upon community ownership, organic TEK formation is possible if HEK can ‘transform’ into LEK over a long period of continued use, increasing its hold in the community. However, with the continued ‘localisation of SEK’ to revalidate HEK, THEK is used here to distinguish indigenous TEK from HEK ‘institutionalisation’. Still, this dynamic exchange of knowledge requires a networking avenue and activities among knowledge holders (Dasanayaka and Matsuda, 2022, p. 2). Hence, building social capital among stakeholders involved in ecological conservation and management projects is necessary to facilitate the transfer of knowledge (Inkpen and Tsang, 2005, p. 160) and enhance the long‐term success of the projects (Yildirim, Alpaslan, and Eker, 2021, p. 2637). To identify how social capital and knowledge transfer played a role in the context of mangrove restoration and rehabilitation, we explore next projects in the Philippines.

## 3 A CASE STUDY OF MANGROVE RESTORATION AND REHABILITATION PROJECTS IN THE PHILIPPINES

The factors involved in the successful implementation and long‐term success of mangrove restoration and rehabilitation projects are identified through an examination of policy documents and project reports of the different organisations and government agencies of the Philippines. A literature search on the Web of Science platform using the keywords ‘mangrove restoration AND Philippines OR ALL mangrove rehabilitation AND Philippines’ and 2000–22 as the publication years was also carried out to supplement further the evaluation. The keywords are applied to a search of publications with topics under both ‘mangrove restoration’ and ‘Philippines’ and ‘mangrove rehabilitation’ and ‘Philippines’. The literature search indicated 68 publications across 25 categories.^1^ The publications were further analysed for their content, with related literature selected that addresses the aim of this study.

### 3.1 Study site

**FIGURE 2:**
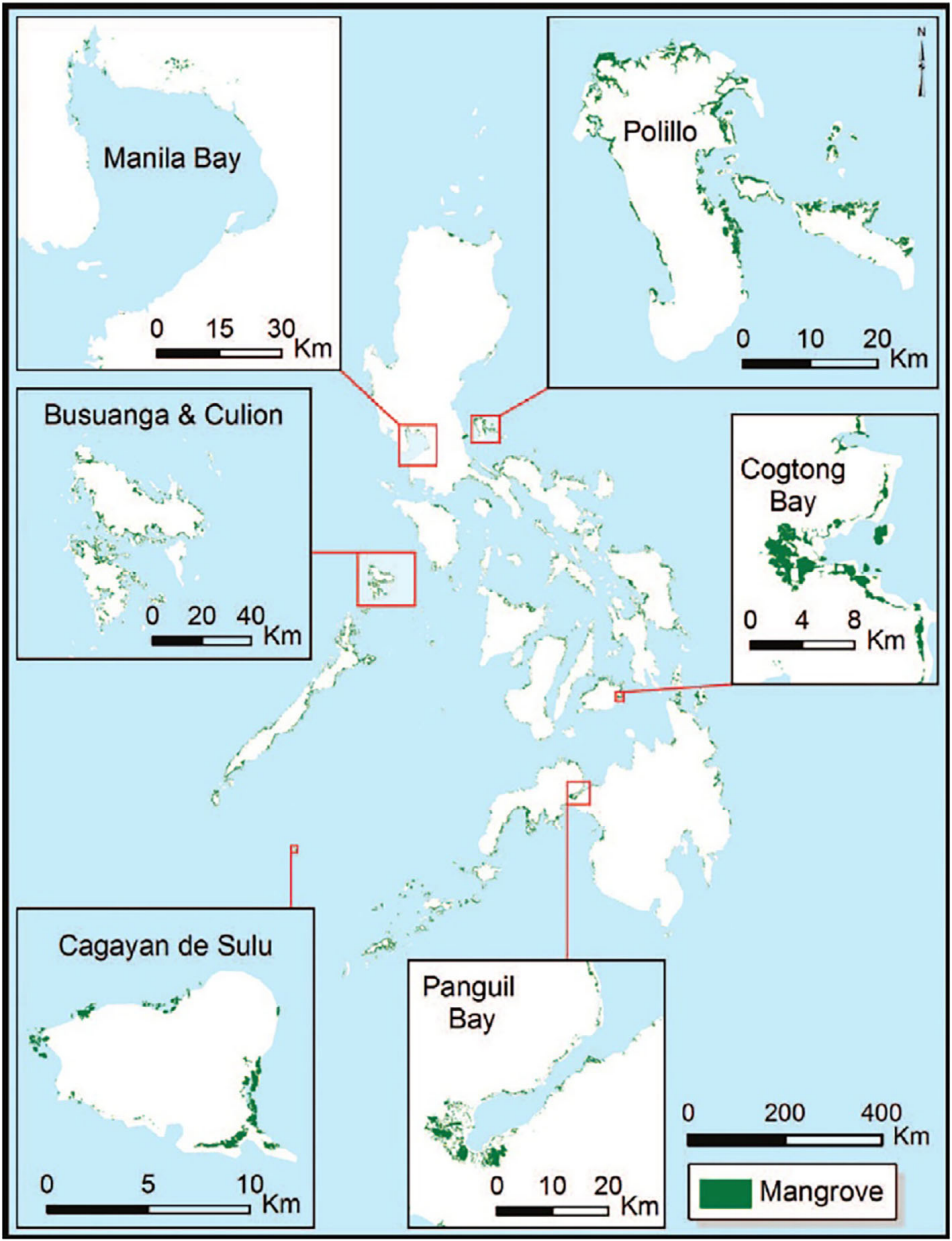
Spatial distribution of mangrove forests in the Philippines in 2010. **Note:** the mangrove forest is represented by green shading on the map. **Source:** reproduced with the permission of the Coastal Education and Research Foundation, Inc., and based on the study of Long et al. (2014, p. 266).

The Philippines is composed of 7,641 islands which are divided into the three island groups of Luzon, Mindanao, and Visayas. Along the coast of the archipelago, diverse mangrove biodiversity is still distributed throughout the country (see Figure 2) despite deforestation (Long et al., 2014, pp. 261, 266). While mangrove reforestation projects have been initiated from as early as the 1980s, an estimated average net loss was continuously reported until 2010 (Long et al., 2014, p. 269). This shows that many mangrove restoration and rehabilitation projects are simply ineffective and have often failed (Primavera and Esteban, 2008, p. 354; Camacho et al., 2020, p. 21).

Philippine government policy mandates the restoration and rehabilitation of mangrove forests. Planning and implementation are led by the Department of Environment and Natural Resources (DENR), a national government agency under the executive branch of the central government. The DENR was authorised to reclaim unproductive, denuded, and degraded national forestlands under the National Greening Program in 2011 (Executive Order No. 26) and the Expanded National Greening Program in 2015 (Executive Order No. 193; DENR, 2019). In support of its mandate, the DENR identified mangrove as one of the priority commodity plants in its Administrative Order No. 2017–03, released on 15 February 2017 (DENR, 2017). This means that mangrove restoration and rehabilitation will be performed to drive economic development while being employed as a strategy in disaster risk reduction vis‐à‐vis the impacts of climate change.

With this kind of priority approach, as well as the lack of trained mangrove rehabilitation specialist among the DENR's project managers in the early years (Primavera, 2015), many of the implementing institutions that received major funding failed to deliver successful projects (Primavera and Esteban, 2008, p. 354). An example of this is the monetary incentivisation of mangrove planting (funded by the national government agency) without SEK planning, which led to the planting of wrong mangrove species in incorrect areas, termed ‘convenient planting’. This resulted in the low survivability of planted seedlings and mangroves overrunning other coastal ecosystems, such as seagrass beds, sand flats, and mudflats. While the survival rate of mangrove plants was low among project sites, continuous replanting was reported because of the cash benefit given for each planted mangrove plant, whether it survives maturity or not (Primavera, 2015; Ranada, 2015). With these ‘convenient’ practices preferred to SEK guidelines and protocols, the many mangrove restoration and rehabilitation projects resulted in either a low mangrove survival rate, which leads to unsuccessful forest restoration, or inappropriate mangrove species propagation, which leads to monospecific plantation (Villamayor et al., 2016, p. 11).

Conversely, many studies have identified the role of LEK and TEK, coupled with SEK, in helping to advance ecological management (Alexander et al., 2019, p. 2; Berkström et al., 2019, p. 2) and formulate inclusive environmental governance (Mistry and Berardi, 2016, p. 2; Beaulieu‐Guay, 2022, p. 404). Exploring the presence of LEK and/or TEK of mangrove ecosystems among participating communities, or the lack thereof, will further help to elucidate the role and use of SEK among local communities. Hence, the following subsection analyses mangrove restoration and rehabilitation projects in the Philippines and how different ecological knowledge is used and transformed during and after project duration.

### 3.2 Social capital and knowledge transfer in mangrove projects

Many mangrove restoration and rehabilitation projects in the Philippines have been funded and implemented by the DENR, with local government unit coordination to facilitate community participation. Significant reforestation and afforestation have been achieved since the 1980s in terms of density, but most projects resulted in monospecific *Rhizophora* plantations (Primavera, 2015; Villamayor et al., 2016, p. 2), based on ‘convenient planting’ rather than SEK. This led to highly dense *Rhizophora* forest throughout the Philippines (including: Olango and Banacon Islands, Bohol; Bantayan Island, Cebu and Tacloban City, Leyte; and Batan Bay Estuary, Aklan), which was reported as cases of ‘successful mangrove restoration and rehabilitation projects’ (Asaeda et al., 2016, p. 59; Villamayor et al., 2016, pp. 5, 8; Ogawa, Resurreccion, and Kanzaki, 2022, p. 2). While forming a thick (one kilometre or more width) and dense (30 trees per 100 square metres) mangrove forest can serve as effective ecosystem‐based protection of the coastline against storm surge and strong winds caused by a typhoon (Sandilyan and Kathiresan, 2015, p. 96; del Valle et al., 2020, p. 269), monospecific *Rhizophora* forests in areas along typhoon pathways have proven ineffective. This was demonstrated by the inability of reforested areas to mitigate the impact of storm surge in Tacloban City (Leyte, Philippines) during Typhoon Haiyan. The vulnerability of a monospecific plantation was further evidenced by the observed high mortality rate of *Rhizophora* as compared with other naturally growing mangrove species endemic to the area (Villamayor et al., 2016, p. 8). The *Rhizophora* mangrove forest is less resilient due to the vulnerability of *Rhizophora* species to strong waves, which is why the Philippine marine scientific community has advised planting *Avicennia* and *Sonneratia* species on the seafront since 2005 and that the monospecific practice be changed to multispecies planting (Primavera and Esteban, 2008, p. 356; Primavera, 2015; Villamayor et al., 2016, p. 12).

Given a high mortality rate and the poor recovery of *Rhizopora*‐dominated mangrove forests whenever a strong typhoon strikes the Philippines, it is to be expected that the long‐term goals of mangrove restoration and rehabilitation projects will not be met. This further emphasises the important role of SEK in ecological management projects. Hence, as part of efforts to address the problem, many mangrove restoration and rehabilitation projects, either funded nationally or internationally, were awarded to SEK‐equipped non‐governmental organisations (NGOs). This provided needed SEK to the project while national government agency personnel are still being trained, and minimised, if not insulated, potential misuse of restoration funds.

However, looking back at the case of the DENR‐funded restoration and conservation mangrove project in New Buswang, Kalibo, Aklan (Philippines) in 1990, which was initiated by the then local government leader, Mayor Allen Quimpo, the selection of the area to be restored was based on a local historian's account, which pointed out that it was once a mangrove forest (Aguirre, 2020). Despite the use of a similar monetary incentive scheme and the lack of an SEK protocol at the start, the mangrove forests grew from 50 to 220 hectares (as of 2020). In this case, the identification of an appropriate plantation area, guided by a local historian, facilitated the immediate success of reforestation, because of better mangrove survival in 1990. This is despite only planting *Rhizophora* species, which led to a monospecific plantation.

Long‐term success is attributed to the people's organisation, Kalibo Save the Mangrove Association, which was granted (by the DENR in 1994) a 25‐year right to use and manage sustainably restored mangrove forest resources under the Forest Land Management Agreement (converted to the Community‐based Forest Management Agreement in 2000) (Aguirre, 2020; Raga‐as et al., 2022, p. 774). The current resiliency of the mangrove forest, now named Bakhawan Eco‐Park, may be attributed to the expansion of the project since 1990; *Avicennia* and *Sonneratia* mangrove trees were planted in 2010 (Raga‐as et al., 2022, p. 781). The inclusion of multispecies planting, following the call to use an SEK guideline, was slowly adopted and implemented by the people's organisation. Kalibo Save the Mangrove Association demonstrated its capacity to rediscover lost LEK through historical accounts and revalidate it through SEK localisation by integrating multispecies planting in 2010, albeit achieving slow transformation.

Replanting of mangroves, either for further expansion of the protected areas or continuous replacement owing to anthropogenic or typhoon destruction, created an avenue for the people's organisation to revalidate its LEK by ‘localising’ SEK, which led to HEK—by planting local mangrove species other than *Rhizophora* in their area, the more resilient the restored mangrove ecosystem. This resulting HEK has been continuously transmitted through strong and effective management of the people's organisation and is reflected in its successful mangrove conservation activity. HEK formation is an important adaptation strategy amid worsening climate change because it allows for the localisation of relevant SEK that addresses unprecedented weather patterns and unstable environmental conditions, and subsequently preserves the long‐term sustainable economic benefits derived from the forest. Still, the preservation and transmission of HEK is only facilitated by the people's organisation, through which access to the direct economic benefits is established. Although its members are part of the wider local communities around the forest that can help to diffuse HEK, there is a need to ‘institutionalise’ HEK in the surrounding local communities so that a stronger ‘sense’ of community involvement beyond the people's organisation can be developed.

In places where mangrove deforestation was particularly severe, it is expected that LEK of the mangrove ecosystem will also be lost because LEK formation relies on human–environment interaction. With the destruction of the ‘environment’ part of the equation, the generation of LEK of the mangrove ecosystem will be limited and perhaps lost. In combination with poverty and the increasing reach and influence of colonisation and globalisation (Turner, Cuerrier, and Joseph, 2022, pp. 636–637), LEK generation may pitch towards ecological knowledge that accords maximum economic benefits to the community based on the remaining mangrove forest, thereby weakening the long‐term holistic view of LEK of sustainable resource management and utilisation (Turner, Cuerrier, and Joseph, 2022, p. 634). For example, in Eastern Samar (Philippines), available LEK of the mangrove ecosystem is limited to how it is utilised as a source of economically important seafood and materials, and how mangrove forest provides coastal protection (Quevedo, Uchiyama, and Kohsaka, 2020, p. 7). This remaining LEK is preserved to accrue continuous short‐term economic benefits from the forest (Quevedo, Uchiyama, and Kohsaka, 2020, p. 4). However, by implementing environmental restoration and conservation projects in the community, HEK formation through localisation of SEK—LEK serves as a framework to integrate sustainable SEK—can be a way to revitalise or rediscover LEK (Turner, Cuerrier, and Joseph, 2022, p. 636).

Nonetheless, the communities near mangrove forests that were mostly involved in restoration and rehabilitation activities are either living on or below the poverty line (Beck and Lange, 2017). This makes the priority of these communities the fulfilment of their basic needs instead of mangrove protection, especially among those who are living from hand to mouth. Some local communities might further equate the sudden restriction on mangrove utilisation imposed by the conservation project with resource prohibition (Katikiro, Macusi, and Deepananda, 2015, p.225), which could lessen its community acceptance and long‐term success. In the Philippines, the indigenous tradition of ‘*bayanihan*’—a spontaneous act of volunteerism to help those in need without the expectation of reward—is ingrained among Filipinos throughout the archipelago (Ealdama, 2012). This concept is applied towards other people and not the environment, hence its employment in mangrove projects was often redirected at community mobilisation by project implementers, which coincides with the initial aim of the projects. With the localisation of SEK at the start of the projects and the willingness of the local community to participate, the exchange of knowledge during planning and implementation should not only lead to HEK formation, but also to the incorporation of sustainable economic and social benefits that the participating communities can derive from the projects, beyond academic discussion. For instance, while HEK can help with the long‐term rehabilitation of mangrove forest, if it is paired with short‐term cash benefits only, then its grip among participating communities will be weak. However, if it is paired with both a short‐term cash benefit and long‐term potential economic income from wild catch by temporarily reseeding the area with young crabs, shrimps, or fish until sustainable natural recruitment occurs, then the hold of HEK on the people's organisation's members can evolve into a positive feedback loop, wherein the more the members protect and expand the mangrove forest, the more seafood they can get.

Therefore, a concrete conceptual framework for mangrove restoration and rehabilitation projects that goes beyond localised SEK guidelines and protocols, and which incorporates a balanced flow of value among natural, social, and economic capitals, is needed for long‐term resilience and sustainable climate change‐proofing. This is important because mangrove restoration and rehabilitation requires at least 10 years of continued monitoring (Teutli Hernández et al., 2020, pp. 5, 26), while the tenure of actors is temporary. The long‐term success of mangrove restoration and rehabilitation projects once project implementers leave the area relies on the organic participation of local communities. Hence, the following subsection assesses the available frameworks used to implement successful mangrove projects in the Philippines. Moreover, the roles of key actors and their interactions with the different types of ecological knowledge are examined.

### 3.3 Analysing restoration and rehabilitation frameworks

**FIGURE 3:**
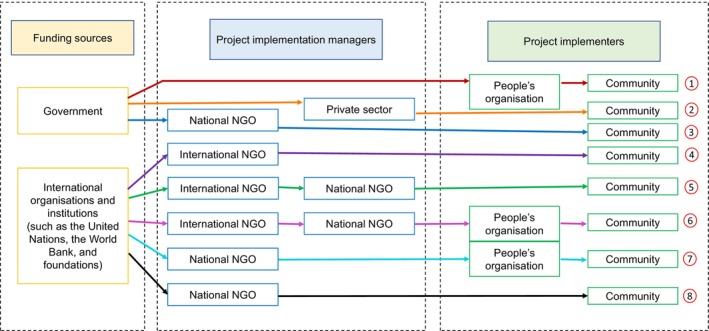
Flowchart showing the mechanisms of rehabilitation activities in Aceh, Indonesia, after the tsunami of 2004. **Source:** authors, based on UNEP (2007, p. 30).

**FIGURE 4:**
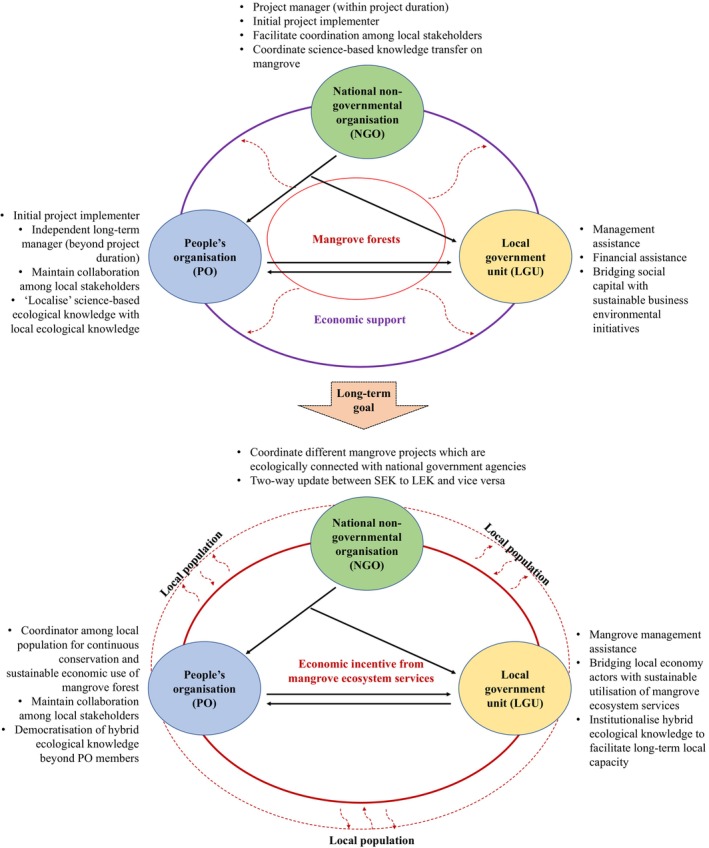
Current and target interactions of key actors in mangrove restoration and rehabilitation project in the Philippines. **Note:** initial implementation (a) and the long‐term goals (b) of mangrove restoration and rehabilitation project show initial interaction among actors (social and economic capitals) in expanding mangrove forests (natural capital) and the evolution of this interaction among actors (social capital) and the mangrove ecosystem (natural and economic capitals), respectively, in the long term. **Source**: authors.

In a report of the United Nations Environment Programme (UNEP, 2007, p. 30), the implementation framework used for the mangrove restoration and rehabilitation projects implemented in Aceh, Indonesia, after the Indian Ocean tsunami of 2004 indicated eight combinations of funding sources, project managers, and project implementers (see Figure 3). Most of the project managers were from national NGOs because of their strong connections with international NGOs from which most of the funds were coming. These national NGOs supervised the implementers, who are part of the local community, in executing mangrove rehabilitation on the ground. The involvement of the implementers was described as ‘cash for work’ because the nature of their participation was based on the workload needed on the ground. The outcome of this approach was high short‐term community participation but a low mangrove survival rate (UNEP, 2007, p. 32), similar to ‘convenient planting’. Likewise, the comprehensive framework developed by Primavera et al. (2012) for the Philippines centred on community‐based mangrove rehabilitation. The only difference between this framework and the former one is the intensive involvement of the community, represented by the people's organisation, with project managers (mostly from national NGOs) present during site selection, planting, maintenance, and monitoring. The science‐based technical guidance and practical training received by the people's organisation during the project period made the community a partner instead of a helper through ‘participatory planning’, ‘participatory implementation’, and ‘participatory monitoring and evaluation’ (Primavera et al., 2012; Camacho et al., 2020, p. 25). However, the limitation of this approach is the long‐term motivation concerning the people's organisation's involvement, which relies on the promise of direct or indirect economic benefits (see Figure 4a) provided by the project (such as ecological services and coastal protection) and partners in government agencies and institutions (such as allowances and cash aids).

Thus, while the community‐based approach was observed to be successful in conveying and contributing HEK, it is recommended that the extent of mangrove restoration and rehabilitation directly coincides with the economic and social benefits that the community can derive (Camacho et al., 2020, p. 25). The long‐term aim is to achieve a robust mangrove ecosystem that can provide economic benefits to the local population (beyond the people's organisation's members) through sustainable utilisation of ecological services, while organically involving the local population in mangrove conservation (see Figure 4b).

Upon further analysis of both frameworks, it was determined that in the Philippines, the three key actors essential for initially implementing projects within the duration of funding are national NGOs, the people's organisation, and the local government unit, while continuous conservation of the mangrove forest to prevent future destruction owing to anthropogenic activities and typhoon impacts relies heavily on the people's organisation with local government unit support. This is on the premise that the national government agency and policy are already mandated and aligned, respectively, with restoration and rehabilitation efforts as seen in the Philippines. The three key actors and corresponding roles and characteristics that are needed for a successful mangrove restoration and rehabilitation project, as suggested by the literature and observed by NGO practitioners, are summarised in Table 1.

**TABLE 1:** The roles and characteristics of essential actors involved in successful mangrove restoration and rehabilitation projects in the Philippines.

Key actors	Roles and characteristics
1. National NGO to which funds had been granted	a. Transfer and implement science‐based knowledge and technical know‐how of mangrove forest restoration and rehabilitation, which are used in target site assessment and project planning and technical training of the people's organisation and the local government unit. b. Coordinate stakeholders at the target site, specifically organising the people's organisation while closely involving the local government unit in the process.
2. People's organisation incubated by the national NGO as the backbone of the project in the community	a. Commit to participation in the project, from planning to implementation (target site assessment and selection, seedling management and planting, and site maintenance and monitoring). b. Undergo technical training in the restoration and rehabilitation of mangrove and receive education in ecosystem service opportunities, for the people's organisation's members.
3. Coastal local government unit where the target site is located	a. Develop own mangrove management plan through a local initiative or as mandated by the Department of the Interior and Local Government, a national government agency governing the local government unit, as part of the requirement for their Seal of Good Local Governance. b. Be overseen by permanent personnel in the Municipal Environment and Natural Resources Office, Municipal Agriculture Office, or the Fisheries and Coastal Resource Management Office, who can be trained as local government unit technical experts. Training can be conducted together with the people's organisation or by an NGO. c. Provide support to the people's organisation in terms of a supervisory role in mangrove monitoring and conservation after the NGO's project period. d. Develop a sustainable economic plan for the people's organisation's members that sustainably utilises the ecosystem services provided by the mangrove project. e. Allocate an annual budget for mangrove rehabilitation and conservation activities based on the local mangrove management plan and sustainable economic plans developed with the people's organisation and NGOs. The budget should include an allocation for science‐based training and monthly operations of the people's organisation to ensure the continuous tenure of informed people's organisation members. f. Attract outside support either for funding or livelihood projects aligned with the mangrove ecosystem services.

**Source:** authors.

**FIGURE 5:**
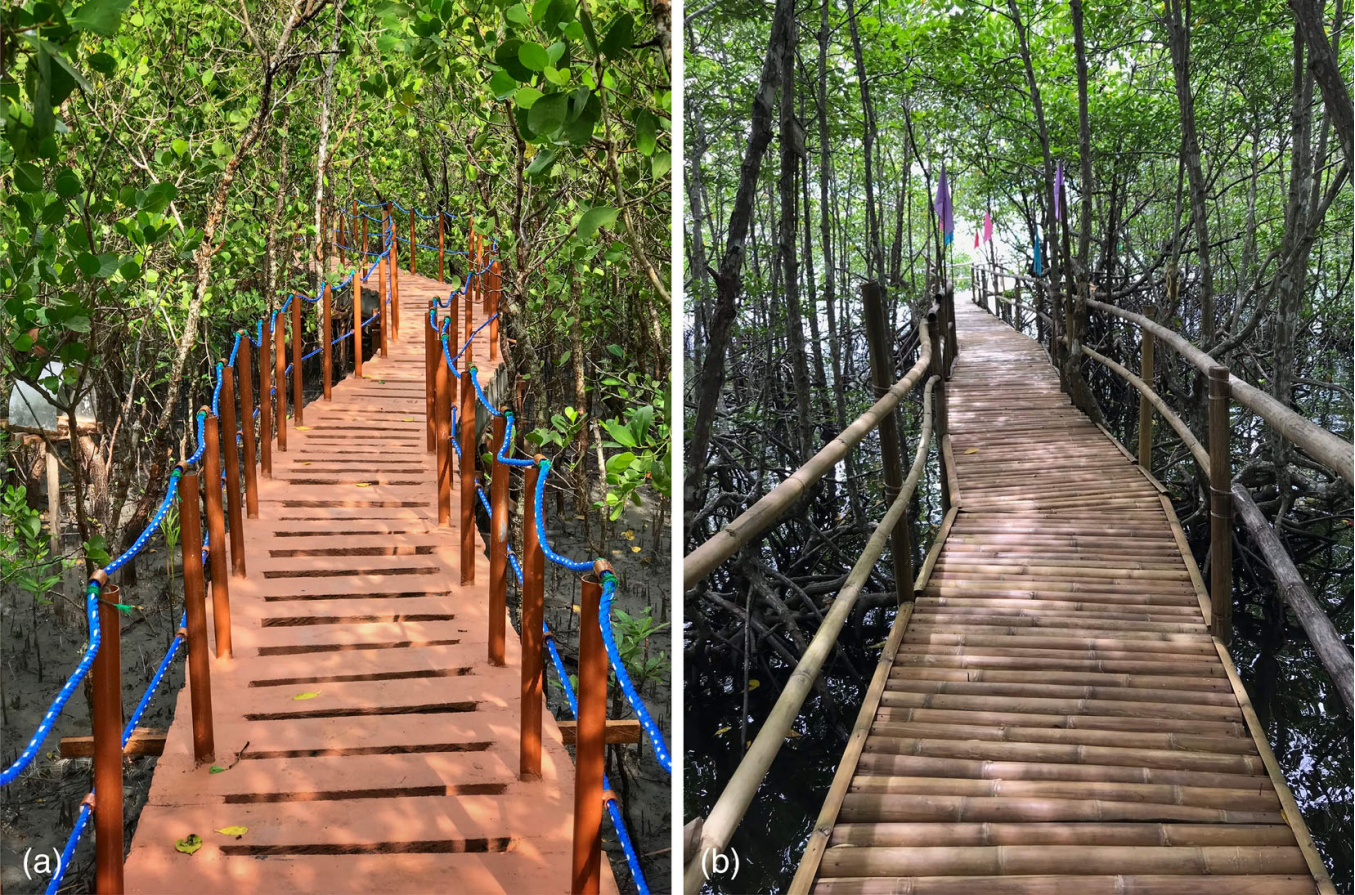
Mangrove forests in Oriental Mindoro, Philippines. **Note:** the figure shows the (a) Silonay Mangrove Conservation Eco‐park in Calapan City and the (b) Puerto Galera Mangrove Conservation and Ecotourism Area. Both have elevated pathways or boardwalks for income‐generating ecotourism activities, which are operated by the people's organisation. These structures bring an overflow of economic activities beyond mangrove ecosystem services through tourism. The improved accessibility and socioeconomic benefits, in turn, sustain the motivation of locals to protect and manage their mangrove areas better. **Source:** Ronald Dionnie Olavides.

The presence of the people's organisation is necessary to anchor directly the transmission and transaction of HEK of mangrove conservation among participatory members. This can be facilitated by the direct short‐term and indirect long‐term economic benefits brought and promised, respectively, by the project. But the high penetration of market integration and globalisation among the neighbouring local communities, in combination with poverty, may present a strong temptation for short‐term gains through unsustainable resource exploitation despite the presence of the people's organisation. To counter this, stronger social ties with nature or ecocultural diversity should be developed. This can be achieved by the transmission of the people's organisation's HEK of sustainable utilisation of the mangrove ecosystem and its coastal protection to the surrounding local population. Subsequently, the transmitted HEK can be ‘institutionalised’ through either organic formation of THEK or ‘facilitated institutionalisation’ through HEK's integration into the local school curriculum (such as mangrove benefits as part of a lesson, science project competition on the importance of mangrove, or children's folktales about mangrove and mystical creatures) and community income‐generating activities (such as social entrepreneurship in ecological aquaculture, creation of annual firefly or mangrove festival events, or the incorporation of ecotourism activities) as coordinated and substantiated by a partner local government unit (see Figure 5). This may lead to the organic involvement of the local population in mangrove conservation by yielding transformative change of their perspective on mangrove projects, from ‘a source of income because of being funded’ and ‘a source of protection and long‐term income from mangrove ecological services’ to ‘a part of their ecocultural identity where its protection equates with social stability’. The renewed involvement can provide experiences to the local population that can further contribute to the rediscovery of LEK as facilitated by the foundation of HEK (see Figure 4b). Therefore, to enhance the long‐term success of mangrove projects, some recommendations are provided to improve the capacity of each identified key actor (see Table 2).

**TABLE 2:** Recommendations on how to improve the capacity of key actors, which aim to achieve a more balanced flow among social, natural, and economic capitals.

Key actors	Recommendations
1. National NGO to which funds had been granted	a. Capacity‐building of NGO field personnel along with relevant local government unit staff and people's organisation leaders in science‐based knowledge can be achieved through course training. The Zoological Society of London (ZSL) Philippines is one of the leading providers of science‐based training in and workshops on mangrove conservation. Relevant personnel can directly undergo training with the ZSL, other trainers who have been trained by the ZSL, or other mangrove experts. b. It is essential for the NGO field personnel to know the local dialect, culture, and community structure. This will enable immersion in the local community and promote community ownership of the project through action with them, not for them.
2. People's organisation incubated by the national NGO as the backbone of the project in the community	a. To build the social capital of the people's organisation with stakeholders, it is important to encourage continuous participation by providing basic economic assistance for the people's organisation's members while passive economic benefits brought by the project cannot yet be felt. This is to offset the immediate opportunity cost (the time and effort taken away by the project) expended by people's organisation's members who are doing voluntary work for the mangrove project. b. Sustainable economic planning can be facilitated by the national NGO but should be developed by the people's organisation in close partnership with the local government unit to allow for independent operation even after the NGO's project period. An example of this is the establishment of an elevated pathway or boardwalk traversing through the mangrove forest (eco‐park), which permits tourists to enjoy the scenery and immerse themselves in nature. Mangrove ranching—where mangrove forest is enclosed with a net for crab, shrimp, or crayfish rearing—can also be integrated into ecotourism activities. For every economic opportunity to be explored, priority should be given to the people's organisation and local stakeholders and equitable benefits should be ensured. This can furnish the people's organisation's members with incentives and can reinforce their commitment to continuous conservation efforts.
3. Coastal local government unit where the target site is located	a. Recognise the benefits of mangrove in economic development and disaster risk preparedness. b. Promote national and local awareness among private businesses and the public with the help of the national government agency. c. Have an open communication with the people's organisation's members and surrounding communities about the project. d. Coordinate with other national government agencies such as the Department of Science and Technology, Department of Agriculture, and the Department of Trade and Industry, which have the practical know‐how and R&D (research and development) on innovative mangrove‐friendly resource utilisation that is beyond conventional means. e. Recognise the potential economic return of the mangrove project, not the cost, through sustainable economic plans and partnership with the private sector.

**Source:** authors.

Moreover, while the flexibility of national NGOs in adapting various situations and conditions in the field made them one of the key actors, it is important to note that the presence of many NGOs implementing the same project in different areas is a sign of inadequate governance. Hence, this is an important indicator for identifying which national government agency and corresponding technical capabilities need improvement. Determining and enhancing the capacity of the concerned national government agency will play an important part in creating an effective inter‐agency policy based on science‐based evidence and systems thinking, where ‘the tipping point, interconnectedness, and resilience’ of the natural, economic, and social aspects of a project are well‐considered (Hynes, Lees, and Müller, 2020). The necessity of an inter‐agency policy for addressing long‐term projects centres on sustainability governance, which requires strong interaction and collaboration across national government agencies (National Research Council, 2013, p. 8). This is especially the case for projects such as mangrove restoration and rehabilitation because the long‐term engagement of multiple stakeholders and continuous monitoring beyond the project site are critical under unstable climatic conditions.

## 4 CONCLUSION

Ecosystem‐based disaster risk reduction to counter the impacts of typhoons in the Philippines should rely on robust and diverse mangrove forest. Attaining this goal depends on successful implementation and continuous conservation of the mangrove ecosystem beyond natural protection, wherein economic and social factors pertaining to local stakeholders are considered.

Three key actors were identified in successful mangrove restoration and rehabilitation projects in the Philippines: national NGOs; the people's organisation; and the local government unit. The use and transfer of SEK by project implementers (in most cases, the national NGO) to both the people's organisation and the local government unit, while considering available LEK, were determined to be important in the initial implementation and long‐term foundation of the mangrove project. While the direct or indirect participation of the local population as either members or non‐members of the people's organisation are necessary for the long‐term conservation of the mangrove ecosystem, the people's organisation is necessary to facilitate the ‘localisation’ of SEK, the rediscovery of LEK, and the organic formation of HEK. The exchange and transfer of this knowledge beyond members of the people's organisation are necessary for building local capacity, empowering the community, instilling community ownership, and promoting a sustainable local economy. HEK formation and transmission should not be exclusive to people's organisation members or the immediate surrounding community, but also include all populations in the locality. In the absence of TEK, continuous local government unit support is needed to ‘institutionalise’ and spread HEK awareness and to infuse its importance in the local population living outside of the project site and across generations. Expansion of the project by attracting additional funding from interested businesses with environmental initiatives, as well as the incorporation of the ecological services that the mangrove ecosystem can provide within the local sustainable economic development framework, can also be accomplished by the local government unit.

As the mangrove ecosystem continuously supplies natural resources and protection for the local population, renewed interaction with nature by locals will help to reestablish and rediscover the LEK of the locality and may lead to TEK or THEK. Moreover, the increasing and cyclical threats of typhoons may serve as a regular reminder to the local population of the importance of continuously protecting the mangrove ecosystem. A typhoon is also a unifying common enemy that can bring locals together to work towards the common objective of helping each other through the long‐term conservation of mangrove forest.

## ETHICS STATEMENT

This paper reports analysis of secondary data.

## Data Availability

The data that support the findings of this study are available from the corresponding author upon reasonable request.

## Endnote

^1^
The 25 categories are primarily environmental sciences (59 per cent), ecology (25 per cent), marine freshwater biology (25 per cent), oceanography (19 per cent), water resources (15 per cent), engineering environmental (10 per cent), biodiversity conservation (7 per cent), environmental studies (7 per cent), fisheries (6 per cent), and geosciences multidisciplinary (6 per cent).
